# Step-by-Step Analysis of a Copper-Mediated Surface-Initiated Atom-Transfer Radical Polymerization Process for Polyacrylamide Brush Synthesis Through Infrared Spectroscopy and Contact Angle Measurements

**DOI:** 10.3390/polym17131835

**Published:** 2025-06-30

**Authors:** Leonardo A. Beneditt-Jimenez, Isidro Cruz-Cruz, Nicolás A. Ulloa-Castillo, Alan O. Sustaita-Narváez

**Affiliations:** 1 School of Engineering and Sciences, Tecnologico de Monterrey, Ave. Eugenio Garza Sada Sur 2501, Monterrey 64840, NL, Mexico; leo.beneditt@tec.mx; 2 Institute of Advanced Materials for Sustainable Manufacturing, Tecnologico de Monterrey, Ave. Eugenio Garza Sada Sur 2501, Monterrey 64840, NL, Mexico; isidro.cruz@tec.mx; 3 Center for Innovation in Digital Technologies, School of Engineering and Sciences, Tecnologico de Monterrey, Ave. Eugenio Garza Sada Sur 2501, Monterrey 64849, NL, Mexico; nicolas.ulloa@tec.mx

**Keywords:** polyacrylamide, polymer brushes, Cu^0^-SI-ATRP synthesis, surface functionalization, parameter interdependency, layer-by-layer analysis, reproducibility in synthesis

## Abstract

Polymer brushes (PBs) are transformative surface-modifying nanostructures, yet their synthesis via controlled methods like copper-mediated surface-initiated atom-transfer radical polymerization (Cu^0^-SI-ATRP) faces reproducibility challenges due to a lack of understanding of parameter interdependencies. This study systematically evaluates the Cu^0^-SI-ATRP process for polyacrylamide brushes (PAM-PBs), aiming to clarify key parameters that influence the synthesis process. This evaluation followed a step-by-step characterization that tracked molecular changes through infrared spectroscopy (IR) and surface development by contact angle (CA) through two different mixing methods: ultrasonic mixing and process simplification (Method A) and following literature-based parameters (Method B). Both methods, consisting of surface activation, 3-aminopropyltriethoxysilane (APTES) deposition, bromoisobutyryl bromide (BiBB) anchoring, and polymerization, were analyzed by varying parameters like concentration, temperature, and time. Results showed ultrasonication during surface activation enhanced siloxane (1139→1115 cm^−1^) and amine (1531 cm^−1^) group availability while reducing APTES concentration to 1 Vol% without drying sufficed for BiBB anchoring. BiBB exhibited insensitivity to concentration but benefited from premixing, evidenced by sharp C–Br (~1170 cm^−1^) and methyl (3000–2800 cm^−1^) bands. Additionally, it was observed that PAM-PBs improved with Method A, which had reduced variance in polymer fingerprint regions compared to Method B. Adding to the above, CA measurements gave complementary step-by-step information along the modifications of the surface, revealing distinct wettability behaviors between bulk PAM and synthesized PAM-PBs (from 51° to 37°). As such, this work identifies key parameter influence (e.g., mixing, BiBB concentration), simplifies steps (drying omission, lower APTES concentration), and demonstrates a step-by-step, systematic parameter decoupling that reduces variability. In essence, this detailed parameter analysis addresses the PAM-PBs synthesis process with better reproducibility than the previously reported synthesis method and achieves the identification of characteristic behaviors across the step-by-step process without the imperative need for higher-cost characterizations.

## 1. Introduction

Material science stands as the cornerstone of technological innovation because it enables solutions to global challenges through the development of novel materials, such as alloys, biomimetic materials, hybrid composites, and stimuli-responsive systems, for tailored functionalities [1]. Among these, polymers have emerged as a transformative class due to their versatility, scalability, and adaptability [2], evolving from traditional uses like packaging or electrical/thermal insulation to multifunctional applications in multifaceted materials, as in polymer-based smart devices [3]. This evolution is closely tied to progress and innovation in synthetic methodologies, where the development of more controlled methods has opened new possibilities.

Controlled polymerization techniques now enable precise manipulation of molecular weight, architecture, and composition, unlocking polymers with programmable behaviors [4,5]. The search for innovations has catalyzed the development of polymer brushes (PBs)— nanostructured materials where polymer chains are tethered to substrates with a high surface density [6]—due to their ability to modify surface properties without altering bulk material characteristics [7,8]. Some applications of PBs include medical solutions, microelectronics, environmental sensors, and surface engineering, among many others [9,10,11,12]. However, challenges persist in synthesis methods for industrial use due to their lack of reliability when they are applied to PBs [13,14,15]. As such, the synthesis of polymer brushes typically involves three stages: substrate functionalization with initiators, surface-initiated polymerization, and post-polymerization modifications [16,17], which demand rigorous control over grafting density and chain length dispersity [18,19]. These challenges have spurred the refinement of synthesis techniques like atom-transfer radical polymerization (ATRP) [20,21], which has allowed precise control over PBs architecture and its physicochemical properties by mitigating premature termination reactions, allowing the synthesis of block copolymers, star-shaped polymers, and hyperbranched structures [22,23,24]. The pursuit to further improve and control architecture and tailor properties led to the development of focused graft-from methods like surface-initiated ATRP (SI-ATRP), which allowed the growth of polymer chains from surface-bound initiators, offering further control over brush density and polymer chain length. Additionally, the use of transition metal catalysts (e.g., Cu^+^, Fe^+^, Ni^+^) offered the ability to mediate radical growth, ensuring low polydispersity and high end-group fidelity [25,26,27]. Despite these advances, the reproducibility in the synthesis of PBs to form thin films remains elusive due to a lack of complete understanding of every step of the process in current methods [28,29].

Polyacrylamide (PAM), a water-soluble polymer, inspired the development of acrylamide-based polymer brushes due to their potential applications in enhanced oil recovery, hydrogel fabrication, biomedical engineering, surface engineering, microsensors, prevention of biofouling, and wastewater treatment [30,31,32,33]. However, the synthesis of PAM polymer brushes faces challenges in polymer conformation, film architecture, and mechanical robustness, leading to the advent of Cu^0^-mediated SI-ATRP (Cu^0^-SI-ATRP), i.e., an SI-ATRP method that uses copper as a metal mediator [34]. This method significantly lowered catalyst concentrations and mitigated oxidative side reactions that introduced variation in the resulting polymerization [34,35]. In addition, this synthesis process enhances industrial viability [36] because it can be performed under ambient conditions, is compatible with aqueous media without compromising synthesis integrity, and enables the deposition of ultra-thick brushes (≥1 μm) with uniform deposition thickness and controlled polymer chain length [37,38,39]. However, challenges remain due to persistent drawbacks in the Cu^0^-SI-ATRP process, including a lack of clarity on the effect of the surface preparation layers on the synthesis of PBs, an absence of standardized substrate preparation steps, and a lack of reproducible results in surface formations [33,40,41].

With the aim of improving the reproducibility of the Cu^0^-SI-ATRP synthesis process, in this study, a systematic step-by-step analysis was conducted to define and clarify the impact of selected parameters on polymer brush formation. This approach aimed at understanding how these parameters hold interdependency from each other and how this affects overall polymer brush synthesis. By decoupling interdependent parameters, this study establishes a framework for reducing batch-to-batch variability. Ex situ step-by-step analysis using Fourier-Transform Infrared Spectroscopy (FTIR) provided molecular-level insights into the progression of the synthesis process, confirming successful initiator immobilization, polymerization, and relevance of some synthesis parameters. In addition, contact angle (CA) measurements allowed for assessing overall surface properties and mapping the uniformity of surface covering through multi-point droplet analysis. Reducing CA from 51° on pristine substrates to 37° after polymer grafting confirmed enhanced wettability. In addition, low standard deviations across measurement sites seem to indicate uniform brush coverage, which is critical for applications requiring interfacial control.

## 2. Materials and Methods

### 2.1. Materials

Acrylamide (AM, electrophoresis grade ≥ 99%), (3-Aminopropyl) triethoxysilane (APTES, 99%), methanol (MeOH, ACS reagent ≥ 99.8%), 2-Bromo-2-methylpropionyl bromide (BIBB, 98%), triethylamine (TEA, ≥99.5%), N,N,N′,N′′,N′′-pentamethyldiethylenetriamine (PMDETA, 99%), polyacrylamide (PAM, 40kMn), and anhydrous tetrahydrofuran (THF, ≥99.9% inhibitor-free) were obtained from Sigma-Aldrich (Naucalpan de Juarez, México). Ethyl alcohol (EtOH, anhydrous, ≥99.5%), hydrochloric acid (36–38%), and distilled water were obtained from JALMEK (San Nicolás de los Garza, México). Silicon wafers were used as substrates with dimensions of 15 × 10 mm^2^. As metal mediators, 0.2 mm thick C1100 copper plates from Easycargo (Ciudad de México, México) were used. Spacers and holders were 3D printed out of polyethylene terephthalate glycol (PETG) obtained from Creality (Shenzhen, China). The anhydrous grade for some chemicals was used here as per availability, although most processes involved water washes and ambient conditions.

### 2.2. Methodology

**Figure 1:**
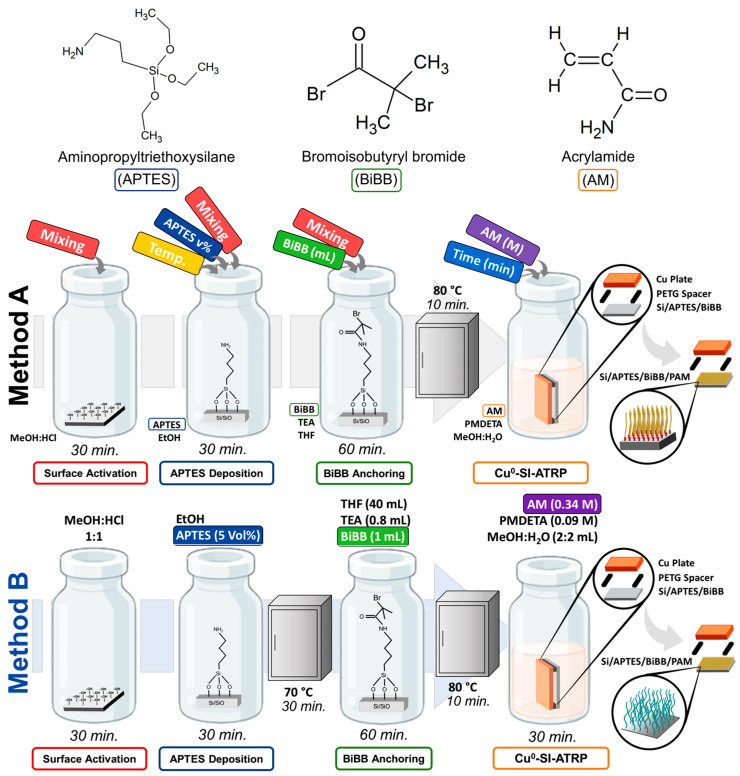
Schematic representation of Cu^0^-SI-ATRP process for the synthesis of PAM-PBs for Method A and Method B.

The experimental process for the deposition of PBs on silicon substrates is schematically depicted in Figure 1. For surface activation, silicon substrates were subjected to three different soaking procedures in a 1:1 (*v*/*v*) MeOH:HCl solution for 30 min: without (*w*/*o*) mixing, under magnetic stirring (MS), and in an ultrasonic bath (US). Next, the wet-etch process was stopped by flooding the container with distilled water and flushing it for 5 min. This was followed by rinsing and flushing with MeOH. Finally, EtOH was introduced to cover the substrates, and they were rested for 5 min. The container with Si substrates was flushed, and fresh ethanol was added to cover the substrates.

For the deposition of the APTES layer, specific amounts of APTES were dropped into a container using a syringe to obtain one of the three concentrations in ethanol: 1 Vol%, 3 Vol%, and 5 Vol%. This deposition step was conducted at two solution temperatures: room temperature (RT) and 60 °C. Previous studies indicated that higher temperatures helped stabilize and accelerate the process [42]. APTES deposition was carried out for 30 min without (*w*/*o*) a mixing process, under magnetic stirring, or in an ultrasonic bath (US). Subsequently, the silicon/APTES samples were rinsed with EtOH and dried under one of the following three conditions: in an oven at 70 °C for 30 min, at room conditions for around 30 min, or simply the substrates were removed from the precursor solution and placed in the following stage (BiBB anchoring) without the drying step.

For the BiBB anchoring over the APTES layer, a container was filled with THF, to which TEA was added. Next, BiBB was then dropped down the walls of the container to obtain a concentration of 40:0.8:x (THF:TEA:BiBB) by volume, where x = 1 stands for the base concentration. Other values of x considered in this study were 0.5 and 2. Since there is no consensus in the scientific literature on the method of mixing at this step [43,44], we investigated the effect on BiBB anchoring. Substrates were introduced into the BiBB solutions without mixing, under magnetic stirring, and in an ultrasonic bath for 60 min. Additionally, other substrates were introduced into premixed BiBB solutions (which had been mixed for 5 min) using the same three mixing methods: without (*w*/*o*) mixing, under magnetic stirring, and in an ultrasonic bath. The Si/APTES samples were allowed to rest immersed in the solution for 55 min, resulting in a total immersion time of 60 min. After initiator anchoring, the samples were removed from the BiBB precursor solution and placed in THF to rinse for 1 min to remove any residual reaction products. This rising step was followed by sequential washes with MeOH and distilled water. The Si/APTES/BiBB samples were then dried in an oven at 80 °C for 10 min, allowing for evaporation of any residual reagent.

The Cu pieces were prepared by placing them into a MeOH:HCl solution (1:1, *v*/*v*) for 5 min, followed by sequential washing with distilled water and MeOH, as per previous studies [37]. An aqueous MeOH solution (1:1 *v*/*v*) was used to prepare the ATRP solution by AM (at 0.34 M, 0.17 M, or 0.09 M) and PMDETA (0.001 M). Substrates were placed face to face with the prepared Cu pieces, separated by PETG spacers of 0.50 mm. This setup was vertically submerged in the ATRP solution and allowed to react for 15 min, 30 min, or 60 min. Upon elapsed time, the deposited films were rinsed with water and then with MeOH before being left to dry at room temperature (RT). This synthesis process will be labeled as “Method A”.

As a reference, a general method previously reported in [36], which adapted Cu^0^-SI-ATRP for various monomers, will be considered and denoted here as “Method B”. According to this method, silicon substrates were introduced in a MeOH:HCl (1:1 *v*/*v*) solution for 30 min, washed, and placed in an alcoholic APTES solution (5 Vol%) for 30 min, from which it was removed and dried in an oven at 70 °C for 30 min. Silicon/APTES samples were introduced into the THF:TEA:BiBB solution (40:0.8:1, by volume) for 60 min and dried in an oven at 80 °C for 10 min. The substrate─spacers─Cu plate arrangement was submerged into the ATRP solution (containing AM at a concentration of 0.34 M) for 30 min before washing and left to dry at room temperature.

### 2.3. Characterization

Characterization of PAM-PBs thin films on Si wafer substrates, as well as every deposition layer, were performed on an FTIR spectrometer (PerkinElmer, Frontier 8000, México City, Mexico) in attenuated total reflectance (ATR) mode using a universal ATR accessory with a ZnSe crystal. Measurements were performed in the spectral range 4000–750 cm^−1^ by averaging 128 scans at a 2 cm^−1^ resolution. Spectra shown here were normalized to the most intense band in the spectral region of interest after baseline correction performed directly on the equipment’s software to highlight relative differences. Regarding the criteria for the spectra in the figures shown here, two alternatives were considered depending on the specific step of interest. Thus, for the previous steps of the polymerization process, that is, from surface activation to BiBB anchoring, the spectra shown here are the average of at least 9 spectra per experimental condition. On the other hand, in the case of PAM and PAM-PBs materials, spectra were chosen considering those with the best signal-to-noise ratio, but the statistics were performed considering 16 measurements for every method.

CA tests were performed using Optical Contact Angle goniometer equipment from Dataphysics. For this, 5 to 6 droplets of 5 µL were automatically dispensed (with the help of the OCA21 software) and spaced throughout every sample to allow individual readings for determining the distribution and homogeneity of the PAM-PBs deposition.

## 3. Results

Analysis was performed using UV-Vis and Raman spectroscopies, AFM, and SEM. Based on the Alexander–DeGennes model (AdG) [2], the chain-to-chain distance for PAM can be estimated after considering the monomer molecular structure in the range of a few angstroms; therefore, microscopy techniques are incapable of perceiving or detecting polymer brushes accurately. Additionally, the extensive properties of brushes make their characterization difficult when using conventional spectroscopic techniques. Raman spectroscopy could not detect significant differences in the polymer structure. UV-Vis lacked reproducible results as the wavelength was too large compared to deposition thickness (~150 nm, as determined by AFM), and the technique was not susceptible to grafting variability. Therefore, these characterization techniques did not contribute to establishing conclusive differences associated with the specific synthesis process of PBs in this work, similar to what was reported in the literature [45,46,47]. Thus, these results will not be shown here. In contrast, step-by-step FTIR spectroscopy and CA measurements provided sufficient evidence to obtain new insights into the synthesis of PAM-PBs.

### 3.1. Surface Activation

**Figure 2:**
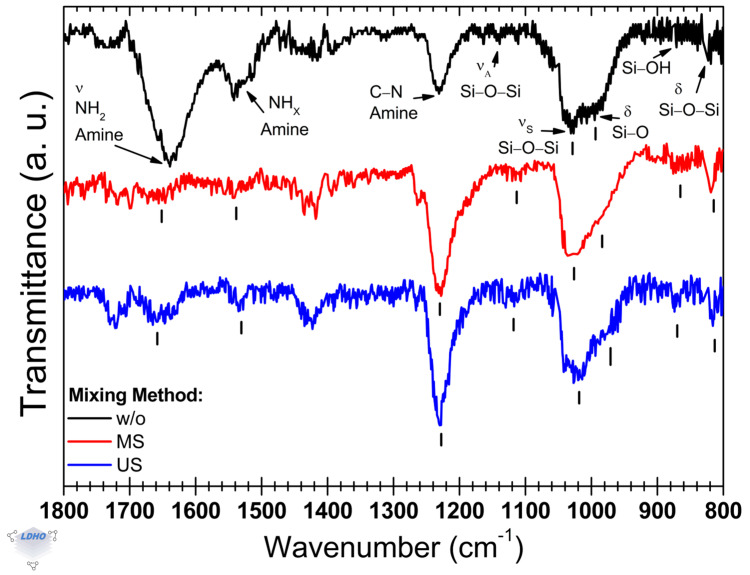
Representative normalized FTIR spectra as obtained after APTES layer deposition on silicon substrates. Surface activation was performed by following one of the three conditions: ultrasonic (US), magnetic stirring (MS), or without any mixing process.

The FTIR spectra in Figure 2 show the vibrational modes obtained after the APTES deposition on silicon substrates. The main functional groups associated with the surface activation are the silanol groups (–Si–OH), which facilitate the anchoring of groups related to the APTES molecule deposited from a solution at a fixed concentration of 5 Vol% in ethanol. The analysis focuses on identifying the amine groups that confirm the successful anchoring of APTES [48,49]. The amine vibrational modes were detected in the spectral range of 1800–1600 cm^−1^ and at 1200 cm^−1^, while the siloxane bands were detected at around 1100 cm^−1^ and at 1000 cm^−1^. The effect of the mixing process during this stage is also discussed.

In the sample without (*w*/*o*) a mixing process, the observed bands associated with siloxane groups are located at 1139 cm^−1^ and 1026 cm^−1^, while the band corresponding to the silanol group is observed at 994 cm^−1^. Additionally, primary amines were identified at 1638 cm^−1^, which corresponds to the most intense band, and secondary amines are present at 1541 cm^−1^. The band at 1231 cm^−1^ is attributed to the C–N stretching of amine. The primary amino band at 1638 cm^−1^ (being *primary_A_1638_* its relative intensity) was found to be approximately 108% more intense than the secondary band at 1541 cm^−1^ (with *secondary_A_1541_* as its relative intensity), resulting in a *primary_A_1638_*/*secondary_A_1541_* ratio of about 2.1. This intensity is also 28% greater than the siloxane band at 1026 cm^−1^. Similar values for this ratio have been reported in other studies, where they were attributed to multilayering [50,51]. Although the exact relationship is unclear, this intensity ratio may be influenced by the orientation of APTES attachment. Furthermore, due to the overlap of the amine bands, this ratio can help to confirm the presence of an end-group primary amine termination (NH_2_) [52,53]. This topic will be explored in greater detail in a later section, but it is worth noting that the primary to secondary amine intensity ratio is crucial in understanding possible explanations related to multilayering, unfavorable attachment, or lack of attachment altogether [42].

The attachment of APTES under magnetic stirring showed differences in the FTIR spectra compared to the deposition process without mixing. In this stirred condition, the amine band observed at 1654 cm^−1^ appears as a shoulder to a smaller band at 1701 cm^−1^, which is attributed to a carboxyl group. This carboxyl band is more pronounced when US agitation is applied. The latter can be related to the unattached siloxane groups with ethoxy terminations [54]. On the other hand, the end-termination of the amine group is characterized by the amine functional groups [53,54], where we calculated a primary-to-secondary amine intensity ratio of 1.6. This ratio is favorable for the subsequent anchoring of the BiBB layer. The results shown in Figure 2 reveal that the most prominent IR band appears at 1230 cm^−1^, with a secondary band at 1031 cm^−1^, attributed to symmetric Si–O–Si stretching, arising when surface activation is conducted under MS. The presence of this band supports the hypothesis of increased amine availability as the surface end-group. Notably, the silanol shoulder associated with the 1031 cm^−1^ band shifts compared to that of a process without any mixing method. Accompanying this shift is a sharper silanol band observed at 806 cm^−1^, which is typically linked to the availability of surface silanol groups [42,52]. Also, the shoulder observed at 995 cm^−1^, for the methodology without mixing (Figure 2, spectrum in black), exhibited a shift to 990 cm^−1^ and a broader width. These findings suggest that there is a reduction in APTES anchorage, as indicated in other studies [55]. Additionally, when the mixing method was utilized, a band with low intensity at 1264 cm^−1^ became present. This band has been attributed to asymmetric stretching of the Si–O–C functional group [42], but this spectral region can also manifest Si-CH_x_ [52] and CH_2_ wagging vibrations [53], all of which are part of APTES backbone propyl chain or ethoxy groups. As the observed behavior of the 1264 cm^−1^ band because of the synthesis process does not contribute to the comprehension of surface coverage or APTES deposition, they will not be considered in this discussion.

On the other hand, when surface activation was performed in an ultrasonic bath (US), its most intense band associated with the amine group of APTES was located at 1233 cm^−1^ (the C–N stretching mode of amine). The asymmetric and symmetric siloxane band positions are centered at 1115 cm^−1^ and 1031 cm^−1^, respectively. These bands, although muddled by noise, had a relative increase in intensity that could be attributed to a higher functional group ordering, possibly caused by favorable and ordered 3-APTES (the tethering of all siloxane-ethoxy chains to the substrate surface) anchorage on the available surface –Si–OH sites, as reported previously [51,55]. This calls for a particular emphasis on the siloxane band at 1115 cm^−1^, as siloxane functional groups are responsible for APTES orientation. Moreover, the band associated with the silanol group is shown as a shoulder that shifted towards 982 cm^−1^ from 994 cm^−1^ (as observed in the black spectrum); the band was shifted by 12 cm^−1^. Additionally, the siloxane bending band at 870 cm^−1^ gained intensity by 90%, along with the silanol band at 809 cm^−1^ gaining intensity by 65%, both compared to the black spectrum in Figure 2. These later bands are generally attributed to the available surface –Si–OH sites, but they could also be related to other vibration modes of –Si–O–Si functional groups. As such, this work notices that incorporating mixing caused siloxane and silanol-attributed bands to have a sharper manifestation and that these may not be exclusive to the surface functional groups but also to APTES molecules with some unattached siloxane groups or multilayering. This interpretation finds ground as the amine bands at 1651 cm^−1^ and at 1531 cm^−1^, respectively, had a reduced *primary_A_1651_*/*secondary_A_1531_* ratio of 1.15 in comparison to the *primary_A_1638_*/*secondary_A_1541_* ratio (=2.1) obtained from FTIR spectrum when no mixing method was used. This is further contrasted by the dominant –C–N amine band (~1233 cm^−1^), which has a relative intensity difference of 87% (*primary_A_1651_*/*secondary_A_1233_* = 7.7). Along with the siloxane band, these bands have been attributed to favorable 3-APTES anchoring by APTES hydrolyzation [42]. The hydrolyzation of APTES causes anchoring where various APTES molecules are connected by siloxane bonds between themselves but leave a siloxane group available for surface tethering. This anchoring process leads to multilayering deposition and is perceived by the manifestation of sharp isolated amine bands [51,52]. In this manner, the FTIR spectrum serves to establish a frame for identifying how APTES is deposited on silicon substrates. Silanol bands with lower ratios to their siloxane bands accentuate a hydrolyzation orientation, with the distinct shoulder at ~980 cm^−1^ being an indicator and siloxane stretching bands at ~1115 cm^−1^ and ~1030 cm^−1^ serving to confirm tethering. Particularly, the asymmetric stretching band at ~1115 cm^−1^ could be the indicator for lateral or hydrolyzed APTES orientation, with ~1030 cm^−1^ siloxane band confirming tethering and the silanol shoulder ~980 cm^−1^ indicating the availability and presence of surface silanol functional groups. Additionally, as observed on the blue spectrum of Figure 2, a *siloxane_1030_*/*silanol_980_* ratio ≥ 1.5 (*w*/*o* = 1.2, MS = 1.5, and US = 1.9) could be distinctive in correlating silanol availability and APTES attachment, with a higher ratio indicating either higher attachment or that the hydrolysis process occurred. Hydrolyzation is not unfavorable, as other works have considered that this process favors homogenous film depositions [42,54].

Through the identification of specific APTES bands to analyze and determine how the deposition and anchoring process is performed, it is possible to discern the effect of three mixing methods on the surface activation of silicon substrates. As APTES was used in this section as an indirect assessment of surface activation, it was found that mixing under the influence of an ultrasonic bath seemed to have a positive effect on possibly more effective film formation. This effectiveness is supported by the obtained *primary_A*/*secondary_A* ratio values (which are affected by the APTES hydrolyzation process), relative siloxane band positions, and *siloxane*/*silanol* ratio values as well. This was not the case for mixing under the influence of a magnetic stirrer, where both lineshape changes and a decreased band intensity suggest a less effective surface coverage or unfavorable APTES deposition. Hence, its implementation in the subsequent deposition layers was not continued. Specifically for the APTES layer, while there is a lack of certainty on monolayer or multilayer formation, the discussion will not focus on it but on favorable APTES anchoring, which will be addressed in the next section.

### 3.2. APTES Deposition

**Figure 3:**
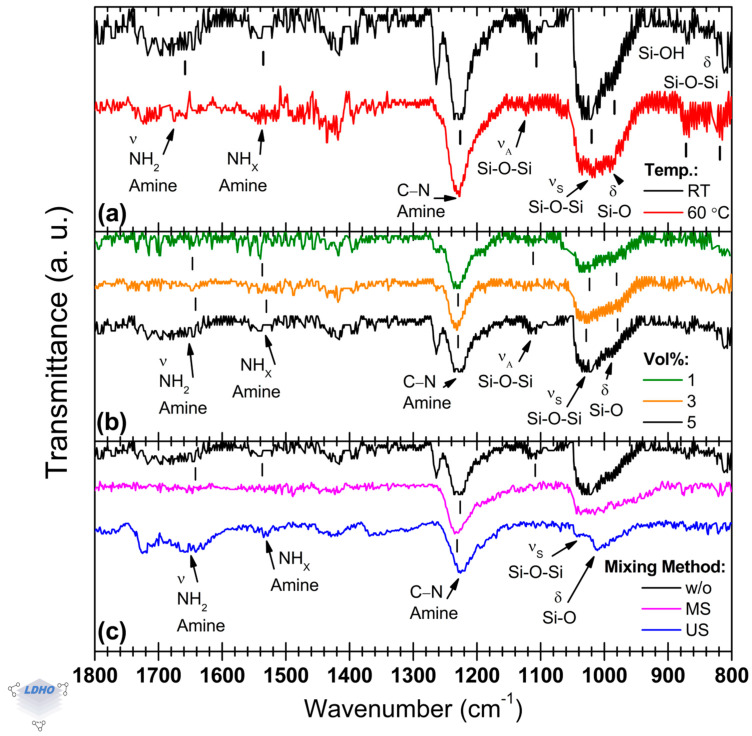
Representative normalized FTIR spectra of APTES deposited on silicon substrates when changing: (**a**) solution temperature, (**b**) APTES concentration, and (**c**) mixing method during deposition.

The main FTIR APTES bands, shown in Figure 3, establish benchmarks for analyzing how APTES is attached to the surface under several synthesis conditions (synthesis temperature, APTES concentration, and mixing method during the process) after surface activation in an ultrasonic bath.

The solution temperature has been reported to benefit APTES monolayer deposition, possibly [42]. However, this was not observed here as both synthesis temperatures, room temperature (RT) and 60 °C, led to similar FTIR spectra, as seen in Figure 3a, with a slight distinction for the spectrum in black (RT conditions) that exhibited the previously mentioned band at ~1260 cm^−1^, which could be attributed to APTES Si–O–C bands and methyl groups present in the molecular backbone. Regardless, as this band has not been found to contribute to discerning the attachment orientation, it can be omitted.

Relative intensities were similar overall, except for the siloxane vibration band at 1113 cm^−1^ for the deposition under RT conditions, which presented a relative intensity increase of 229% over the spectrum for the material deposited from an APTES solution at 60 °C. This band is paired with the siloxane band at 1021 cm^−1^, attributed to siloxane formation from APTES anchoring, which has a similar relative intensity for materials synthesized under both temperature conditions. There is a lack of certainty regarding the assignment of these bands. Some studies, for instance, assign the band located at 1113 cm^−1^ to the contribution coming from siloxane formation, whereas the band at 1021 cm^−1^ has been attributed to linear (also called transversal) siloxanes, which are directly attached to the silicon surface in contrast to lateral siloxanes [53,55]. Therefore, this could imply that APTES attachment through transversal siloxane groups was favored during APTES deposition at room temperature due to more effective surface functionalization. However, quantification of surface functional groups density is a challenge, and at this point, it is not possible to offer additional evidence to support this better effectiveness in surface functionalization by FTIR measurements only. As mentioned in the previous section, the shoulder at ~990 cm^−1^ can be attributed to the Si–O bending mode in the siloxane functional group, which can be differentiated from the silanol vibration mode observed in the spectral region of ~900–800 cm^−1^ belonging to surface-bound functional groups. The samples synthesized at room temperature presented a *siloxane_1021_*/*silanol_992_* ratio of 1.4, while those synthesized at 60 °C had a *siloxane_1019_*/*silanol_991_* ratio of 1.1. It suggests a relatively high density of silanol functional groups, which can be caused by hydrolyzation, unfavorable orientation, or incomplete APTES attachment. However, this spectral region is also susceptible to the presence of surface-bound functional groups, which makes the temperature an inconclusive parameter for the APTES deposition. Despite this, clearer information can be obtained from the observed changes in the FTIR spectra because of the effect of other parameters.

Concerning the effect of APTES concentration (Figure 3b), using FTIR spectroscopy, it was observed that 1 Vol% was enough to ensure surface attachment of APTES. The other concentrations only promoted differences in the relative intensity of the bands associated with amine and siloxane functional groups, which can be attributed to multilayering. The spectra for 1 and 3 Vol% concentrations showed a *primary_A_1652_*/*secondary_A_1535_* ratio of 0.6, i.e., the secondary amine band around 1535 cm^−1^ possesses a higher relative intensity than that corresponding to the primary amine functional group located at 1652 cm^−1^. In addition, at an APTES concentration of 5 Vol%, the dominant amine band was shifted towards 1659 cm^−1^, and the *primary_A_1659_*/*secondary_A_1535_* ratio was modified to 1.6. Despite this observed change in the relative intensities of the bands, the *primary_A*/*secondary_A* ratio offers partial information; therefore, additional information coming from other FTIR bands is necessary as support. For example, despite similarities in their lineshapes among the spectra of Figure 3b, two bands are easily observable at ~1113 cm^−1^ (siloxane) and ~1260 cm^−1^ (previously discussed) at the highest APTES concentration considered here (5 Vol%, black spectrum in Figure 3b). It could be correlated to favorable attachment or APTES multilayer deposition because of the relatively high APTES concentration. In addition, it could be supported by the *amine_1230_*/*siloxane_1025_* ratio (=1.3) for the highest APTES concentration, which also changed compared to the other two conditions (2.2 and 1.7 for the APTES concentrations of 1 and 3 Vol%, respectively).

The mixing method is the parameter that promoted a noticeable difference in FTIR spectra, as seen in Figure 3c. As suggested by the FTIR measurements, depending on the specific mixing method, APTES molecule attachment can be hindered. If APTES deposition is performed without any mixing method (like either US or MS), some bands can be observed at 1652 cm^−1^ (primary amine), 1260 cm^−1^, 1228 cm^−1^ (amine), and at 1025 cm^−1^ (siloxane), as well as the silanol bands at 872 cm^−1^ and 816 cm^−1^. Otherwise, deposition under US or MS mixing methods led to poor reproducibility, and sometimes, it was not possible to observe the whole set of characteristic FTIR bands of the APTES molecule. Thus, it is difficult to confirm how specifically the APTES molecule is attached to the substrate depending on the mixing method, i.e., it is not possible to distinguish between primary orientation, partial reaction (unfavorable), or crosslinking (due to hydrolysis), for example [51,55]. However, based on some differences in the band positions and some modifications in the spectra’s lineshape, it seems that there is sufficient evidence for unfavorable APTES orientation when a mixing method is used. If US is utilized during APTES deposition, for example, the characteristic band for amine is manifested by the convolution of two bands, the first at 1655 cm^−1^ and the second at 1615 cm^−1^. In addition, the siloxane band, which is usually located at ~1100 cm^−1^, is shifted to ~1090 cm^−1^, whereas the band at 1024 cm^−1^ has reduced sharpness and increased FWHM, with a shoulder extending towards 965 cm^−1^.

In summary, although APTES deposition from the precursor solution at a concentration of 1 Vol% was confirmed, the quality of the thin film is unclear. Additionally, mixing methods were counter-productive for a favorable APTES molecule attachment. Therefore, based on FTIR measurements, APTES deposition from a 5 Vol% precursor solution at room temperature without a mixing process seems to be appropriate for further BiBB anchoring.

### 3.3. Effect of the Drying Process on APTES Deposition

**Figure 4:**
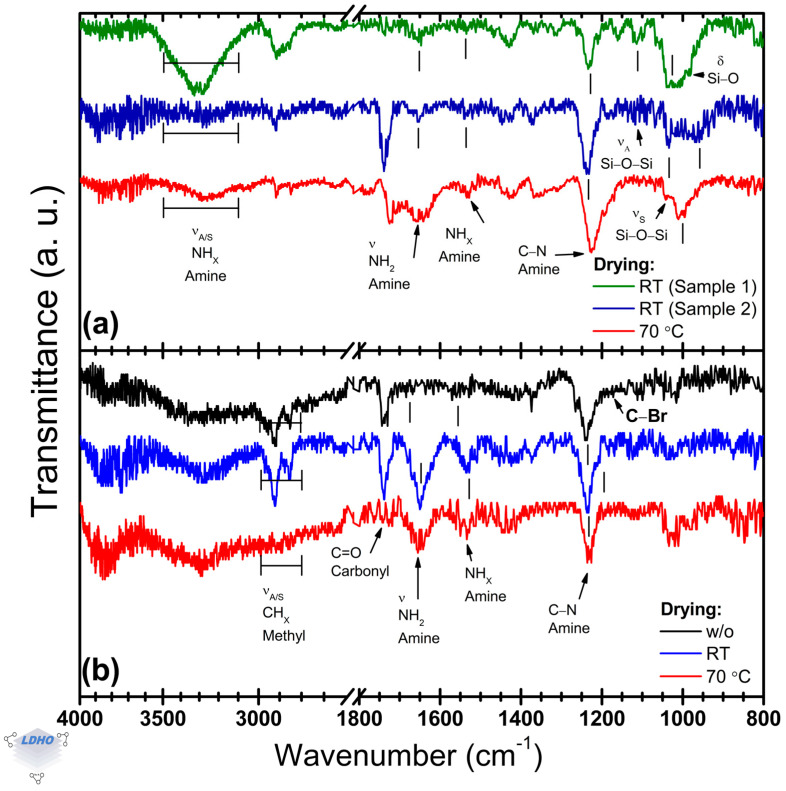
Representative normalized FTIR spectra for APTES thin films dried at two temperatures (**a**) and for BiBB anchored over Si/APTES samples when the APTES layer was dried under two conditions (**b**). In (**a**), spectra for two dried samples at room temperature are shown as evidence of poor reproducibility.

The impact of drying methods on APTES film formation has been previously reported in the scientific literature in the range from 30 to 60 °C but in a different context to PBs synthesis [55]. Figure 4a shows the effect of two drying conditions on the FTIR spectra of APTES thin films. In addition, the effect on BiBB anchoring is also considered as indirect evidence for the APTES drying process (Figure 4b).

Differences have been observed in the amine bands depending on the drying process. At room temperature, the deposited films presented poor reproducibility, as illustrated in Figure 4a. The amine (NH_2_) region at high wavenumbers (3500–3000 cm^−1^) shows poorly defined bands. RT-dried samples could present, or not, two distinct amine bands located at 3332 cm^−1^ and 3277 cm^−1^, which are assigned to asymmetric and symmetric stretching vibrations, respectively. The relative intensity of these bands is around 73% higher than that observed on APTES films dried at 70 °C. These amine bands have not been correlated to film quality or surface APTES orientation in the literature, but the observed difference in the FTIR spectra could be attributed to adhered APTES material or residual water in the material due to their relative intensities.

Independently of the drying conditions, APTES thin films exhibit their bands for primary amine and tertiary amine functional groups at the same position (at 1654 cm^−1^ and 1531 cm^−1^, respectively), but the band position of a carbonyl group was observed for both 70 °C and those dried at RT from possible ethoxy group terminations or trapped ambient CO_2_. Additionally, the *primary_A_1652_*/*secondary_A_1530_* ratio was modified from 2.8 (70 °C) to 1.6 for dried samples at RT. Although the drying process has been considered beneficial in the literature, from Figure 4b, it seems that there are no significant differences in the APTES films’ properties to hinder further BiBB anchoring. Upon the BiBB anchoring, the amine band of the APTES film (initially located at ~1230 cm^−1^) was shifted to ~1245 cm^−1^. Moreover, methyl bands are clearly observed in the 3000–2800 cm^−1^ region due to their increased intensity. It is worth noting that, in comparison to the dried samples, the BiBB samples without any drying process showed a reduction by 73% and 25% in the intensity of their bands attributed to the primary and secondary amine functional groups, respectively. While the presence of secondary amine bands in FTIR spectra is desirable, as they confirm BiBB anchoring, primary amine bands are considered unfavorable as they correlate with exposed end functional groups that were not activated.

With the confirmation of BiBB anchoring, even without any drying process, the requirements for successful BiBB anchorage and adequate surface covering are questioned.

### 3.4. BiBB Anchoring

**Figure 5:**
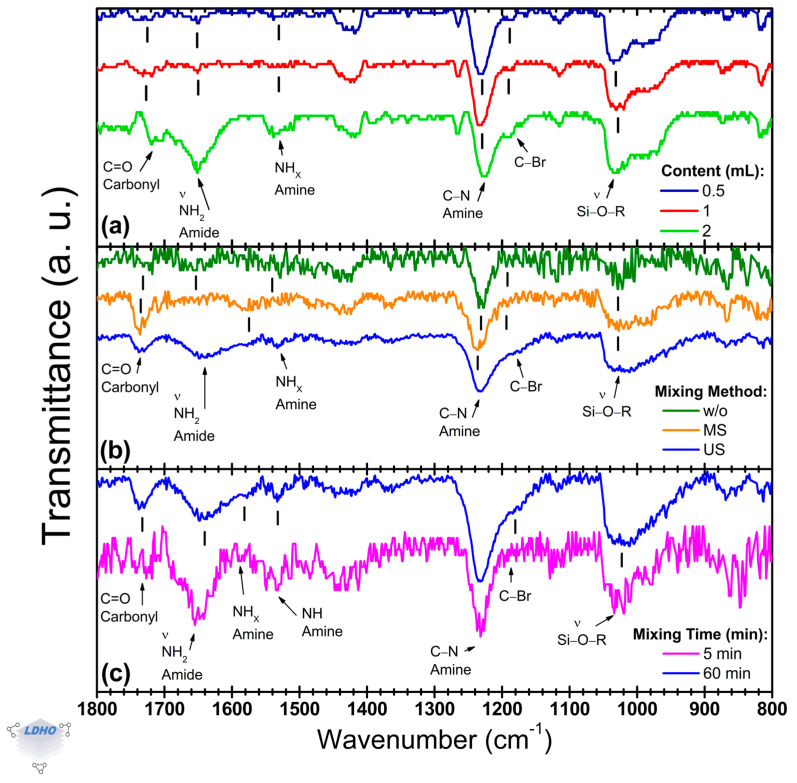
Representative normalized FTIR spectra obtained after BiBB anchoring over Si/APTES samples for different (**a**) BiBB concentrations, (**b**) solution mixing methods, and (**c**) mixing times.

Focusing on BiBB anchoring, Figure 5 presents the FTIR bands used to identify the presence of the initiator. The BiBB anchoring is crucial to improve surface preparation and increase the availability of initiators for the PBs synthesis, by which three parameters were explored: initiator concentration (Figure 5a), mixing method (Figure 5b), and mixing time (Figure 5c).

Previous works have identified the products and by-products of the BiBB solution, as well as how the precursor solution enables BiBB anchoring [56,57,58]. However, it was observed from Section 3.3 that the deposition conditions for the APTES layer had little to no influence on the BiBB anchorage. This could be caused by interactions between the solution by-products and the APTES layer that ensure BiBB anchorage regardless of APTES deposition parameters, but this mechanism is not examined in this work. Regardless, Figure 5 shows bands belonging to Si/APTES and Si/APTES/BiBB samples, with certain bands gaining relevance. Among these, a change in the spectral lineshape is caused by the transition from amine to amide, and a shoulder at ~1170 cm^−1^ is observed among the structural bands. Smith et al. [59] proposed the identification of halogen bands at higher wave numbers by carbon bands such as a C–Br vibrational mode. These vibrational modes were identified in the 1200–1000 cm^−1^ region, overlapping with many other carbon-based bands, making them difficult to identify. However, since the C–Br bond is an end-group termination at the surface level, it could favor their detection, allowing this uncommon band to be identified as confirmation of BiBB anchorage.

By varying the *x* amount of BiBB from 0.5 to 2 mL in the THF:TEA:BiBB precursor solution (40:0.8:*x*, by volume), noticeable changes in the relative intensity of amide, bromidyl, and carbonyl bands were observed (Figure 5a). Increasing the amount of BiBB to 2 mL seemed to cause a change in either the BiBB anchoring or the APTES layer as carbonyl and amide bands convoluted at 1709 cm^−1^ (as a shoulder) and 1651 cm^−1^, respectively, with a relative intensity increase of up to 69%. Additionally, intensity changes were observed in the siloxane band at 1028 cm^−1^ with its silanol shoulder, but neither of these changes has a clear explanation as these bands are mostly attributed to APTES molecule and surface-level interactions [42,52]. However, the manifestation of the C–Br stretch proposed previously [59] was appreciable, becoming more distinct as an independent shoulder at higher concentration. This relative intensity shift may allude to a higher density of initiator availability and supports its correlation to a bromide end-group termination.

The incorporation of mixing methods caused an impact on BiBB anchoring that could be correlated to surface activation, hence molecule end-group fidelity (Figure 5b). Additionally, mixing seems to make the C–Br band at ~1170 cm^−1^ become more noticeable as a shoulder, along with defined sharpness of secondary amide groups tending towards ~1550 cm^−1^ or lower. These bands may correlate with amine site availability and successfully anchored BiB- (activated isopropylbromide by loss of a bromide) molecules. The spectra without mixing differed from mixed sample spectra (Figure 5b), as the former presented a primary amide band at ~1660 cm^−1^, with a secondary amide at ~1585 cm^−1^, and a tertiary amide or amine at ~1530 cm^−1^. Stirred (MS) samples differed as they lacked a distinguishable primary amide band, but spectra for sonicated (US) samples did express amide bands, with some shifts. While these shifts do not change the functional group and molecular vibration interpretation, the primary-to-secondary amide ratio (*primary_Am_1637_*/*secondary_Am_1596_*) and the *primary_Am_1637_*/*secondary_Am_1530_* ratios changed to 1.6 and 2.3, respectively, from without mixing’s ratios of 2.3 and 1.3. This change poses a distinction and possible correlation of bands as amides (primary and secondary) confirm BiBB anchoring but could also allude to brush density [6,60,61]. The reduction from 2.3 to 1.6 may be attributed to hydrogen bonds or lateral pressure from other BiBB groups or amide sites tethering a larger number of BiBB molecules as the secondary amide (NH_x_) stretching vibrational mode is susceptible to these pressures [62,63]. Furthermore, the increase in the relative intensities from 1.3 to 2.3 supports this observation as the tertiary amide, usually non-observed, is identified at lower wavenumbers (~1530 cm^−1^) and may be attributed to amines from APTES (non-activated sites). US mixing seems to reduce the presence of non-activated amine sites, possibly confirming a higher BiBB anchoring density. However, the relationship between the intensities of the amide and amine functional groups is complex and cannot be used as the sole confirmation. As such, the C–Br band at 1177 cm^−1^ is of particular interest as it can be directly correlated to the BiBB molecule. This band manifested in US method spectra as a distinct shoulder, unlike in the stirred method and the method *w*/*o* mixing. Additionally, the C–Br band may not be tied only to BiBB anchorage but also to end group fidelity, complementing the analysis of indirect bands and confirming initiator density.

Expanding on mixing impact in BiBB anchoring, “continuous mixing” (i.e., having Si/APTES samples immersed from the start in the precursor solution) differed from “premixed solutions” (where the Si/APTES samples were immersed into the precursor solution after mixing), as denoted by changes in amide–amine and C–Br bands, with all becoming magnified by premixing (Figure 5c). Deposited samples from premixed precursor solutions had amide bands centered at 1596 cm^−1^ and 1558 cm^−1^ (assigned to secondary amide NH_x_ and tertiary amide, respectively), which could suggest a higher density of activated sites. As the primary amide group at 1641 cm^−1^ can be attributed to an anchored BiBB by the carbonyl overlapping at 1726 cm^−1^, the secondary amide could confirm the activated sites, and a tertiary amide possibly being a consequence of dense surface activation where an amine may be housing more than one BiBB molecule. Additionally, based on the shoulder widening of the C–Br band and the relative increase in its intensity, BiBB anchoring from premixed precursor solutions proved to be effective and could ensure a higher BiBB surface anchorage density. In this context, both mixing methods showed a band attributed to carbonyl at 1726 cm^−1^ to further confirm the amine-to-amide transition by the BiBB anchoring.

### 3.5. Cu^0^-SI-ATRP Difference

**Figure 6:**
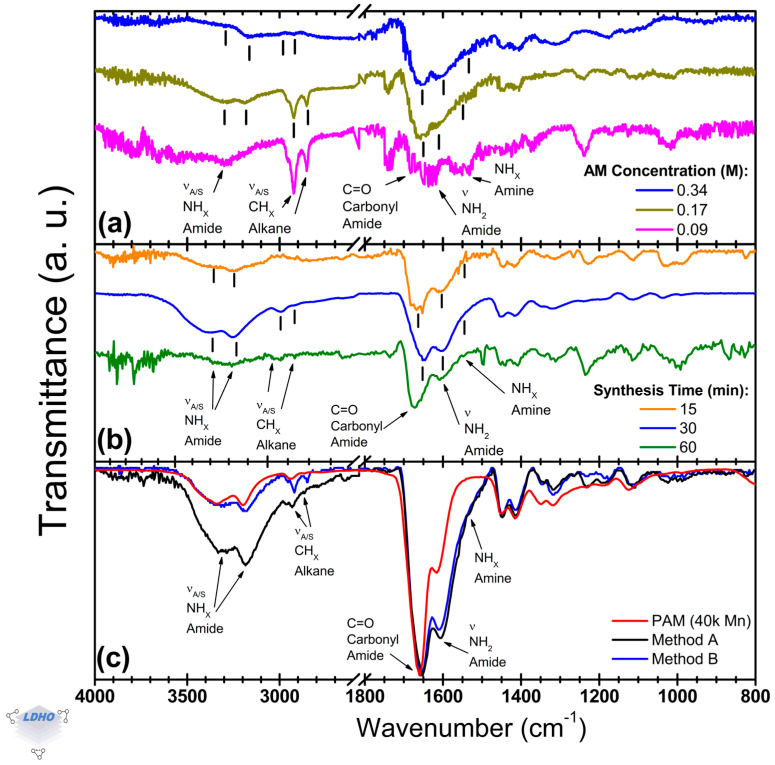
Representative normalized FTIR spectra for Cu^0^-SI-ATRP PAM after changes to (**a**) acrylamide (AM) molar content, (**b**) synthesis time, and (**c**) spectra for commercial PAM. This work’s method (Method A) and a general Cu^0^-SI-ATRP method (Method B) were used to observe similarities and confirm PAM formation.

This section identifies the characteristic bands of PAM and focuses on amide functional group bands and methyl bands, as illustrated in Figure 6. Key parameters, such as the concentration of acrylamide (AM) in the ATRP solution (Figure 6a) and the duration of the synthesis process (Figure 6b), can be evaluated to optimize PAM brush formation via the Cu^0^-SI-ATRP method. Furthermore, utilizing a commercial PAM as a comparative reference allows for a more thorough analysis and comparison of synthesis methods (Figure 6c). Overall, the PAM bands resulting from the Cu^0^-SI-ATRP synthesis were successfully characterized, revealing characteristic amide twin bands at 1656 cm^−1^ and 1610 cm^−1^, confirmation of alkane methyl groups within the 3000–2800 cm^−1^ range, and asymmetric and symmetric amide bands at 3337 cm^−1^ and 3168 cm^−1^, respectively.

The concentration of AM in the solution has an influence on managing the polymerization process. When the amount of AM is reduced to a quarter of the original level (0.09 M), it can lead to erratic polymerization. However, using half of the original concentration (0.17 M, down from 0.34 M) is sufficient to facilitate brush formation (as shown in Figure 6a). Both concentrations produced similar spectral bands for the asymmetric and symmetric amide (at 3326 cm^−1^ and 3183 cm^−1^, respectively), asymmetric and symmetric methyl (2923 cm^−1^ and 2856 cm^−1^), amide–carbonyl group (1645 cm^−1^), primary amide (1603 cm^−1^), and secondary amide (around 1542 cm^−1^). This indicates that similar PAM formations were achieved in both cases, enabling brush synthesis with less material. However, at a concentration of 0.09 M, there was a loss of bands for amide stretches and noticeable shifts in position, which highlighted issues with the control of PAM synthesis, causing possible voids or aggregates on the surface. Specifically, the primary amide band at 1603 cm^−1^ shifted towards the secondary amide region at 1579 cm^−1^, and a tertiary amine band appeared at 1530 cm^−1^. This behavior has been linked in other studies to crosslinking or branching [23,62,64]. While this study did not investigate the chemical balance in the solution that may cause these changes, it is suggested that the lower concentration of AM leads to disordered and non-uniform growth, resulting in the branching of PAM chains.

Synthesis time variations revealed a timeframe for the formation of PAM brushes. The PAM spectra at 15 min and 30 min were similar, but after 30 min, notable deformations in the lineshape indicated excessive and uncontrolled polymerization (see Figure 6b). At 15 min, polymerization was inconsistent; the spectra often lacked evidence of polymerization in the expected amide region (3500–3000 cm^−1^) and the methyl bands (3000–2800 cm^−1^). However, when successful, the 15 min synthesis exhibited distinct bands corresponding to saturated alkanes (1668 cm^−1^), primary amides (1612 cm^−1^), and a shoulder peak indicative of secondary amides (1570 cm^−1^), confirming PAM formation like that observed at 30 min. The inconsistencies noted might relate to the polymerization kinetics involved in the initiation process. At 60 min, the twin bands correspond to the amide-carbonyl, and primary amide began to deteriorate. The *carbonyl_1672_*/*primary_1608_* amide ratio was 1.7, differing from the ratios of 1.2 and 1.4 observed at 30 min (with bands at 1646 cm^−1^ and 1604 cm^−1^) and 15 min, respectively. Previous studies highlighted this relative intensity relationship concerning PAM conformation [64]. While this study notes a correlation between PAM conformation and chain growth, as observed through these functional group bands, further investigation is necessary to confirm these findings. Overall, there appears to be a timeframe for PAM brush formation, where 15 min is the minimum time needed to initiate brush polymerization. Beyond 60 min, the structure may collapse or undergo multilayer deposition or crosslinking, resembling bulk material. This was further investigated by comparing different PAM films, particularly those produced by the proposed Method A, with those from Method B and bulk commercial PAM.

Commercially bought PAM, as compared to PAM brushes synthesized through Method A and Method B, exhibited similar widths and molecular band positions. However, subtle variations were noted at higher wavenumbers in the asymmetric and symmetric amide bands, as well as in the alkane methyl bands. Notably, at higher wavenumbers, there was an intensity reduction of up to 21% compared to the dominant amide-carbonyl C=O band around 1660 cm^−1^ for all samples. The Cu^0^-SI-ATRP methods featured a primary amide band at 1606 cm^−1^, with an amine shoulder near 1560 cm^−1^. In contrast, the *carbonyl_1660_*/*primary_1606_* amide ratio for these methods was 1.2, while for bulk PAM, it was 2.0. While polymerization can be confirmed, these differences between commercial bulk material and the Cu^0^-SI-ATRP methods are attributed to the deposition methods themselves. However, the observed disparities in lineshapes suggest conformational differences among the synthesized materials, which have been addressed in previous works by analyzing the amide functional group bands (their presence or absence in the spectra, position, and relative intensity) in such a way that is possible to determine primary (–CONH_2_), secondary (–CONH–R), or tertiary (–CON–R_2_) amide side-groups [64,65,66].

Considering the spectral region between 3600 and 2600 cm^−1^, significant differences in the amide asymmetric and symmetric contributions were observed, with peaks at 3329 cm^−1^ and 3337 cm^−1^ for samples synthesized following the Method A and at 3181 cm^−1^ and 3168 cm^−1^ for those samples from Method B. However, no significant differences were noted for the asymmetric and symmetric methyl bands. In the amide spectral range (1800–1500 cm^−1^), Welch’s t-test indicated a difference only for the distinct amide carbonyl bands at 1652 cm^−1^ and 1656 cm^−1^ for Method A and Method B.

**Figure 7:**
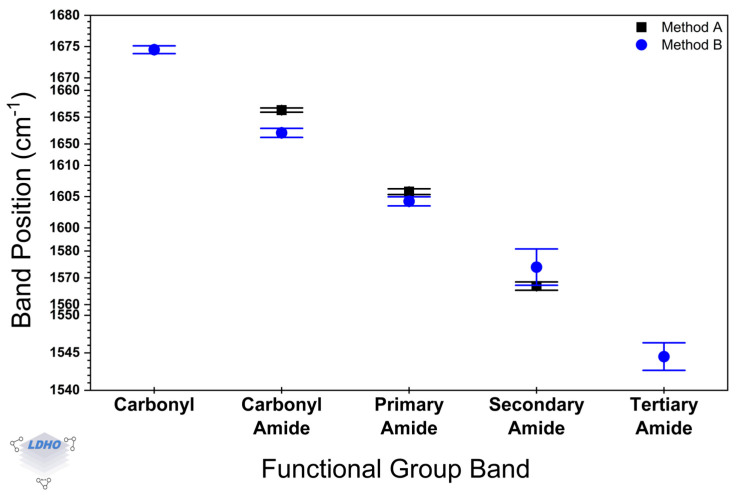
Average peak positions and standard deviation for selected bands in the spectral region of 1800–1500 cm^−1^.

Statistical analysis comparing samples from Method A and Method B revealed differences in repeatability through spectral evaluation (Figure 7). For this, the spectra of 16 samples for every method were considered and deconvoluted into molecular groups, following a method like Murugan et al. [67], using an FWHM approach with Lorentz fitting. Data in Figure 7 illustrate the average peak positions for the functional groups in the spectral region of 1800–1500 cm^−1^, with their corresponding standard deviation. Compared to Method B, Method A exhibits a narrow distribution for the peak positions, which could suggest less variability in the PBs properties when Method A is the chosen synthesis route. In addition, Method A exhibited the expected FTIR bands for the amide functional group in PAM-PBs, whereas Method B exhibited less common additional bands, which can be attributed to some irregularities in the material (like branching or crosslinking) during the polymerization process.

### 3.6. Effect on the Macro-Scale: Contact Angle

**Figure 8:**
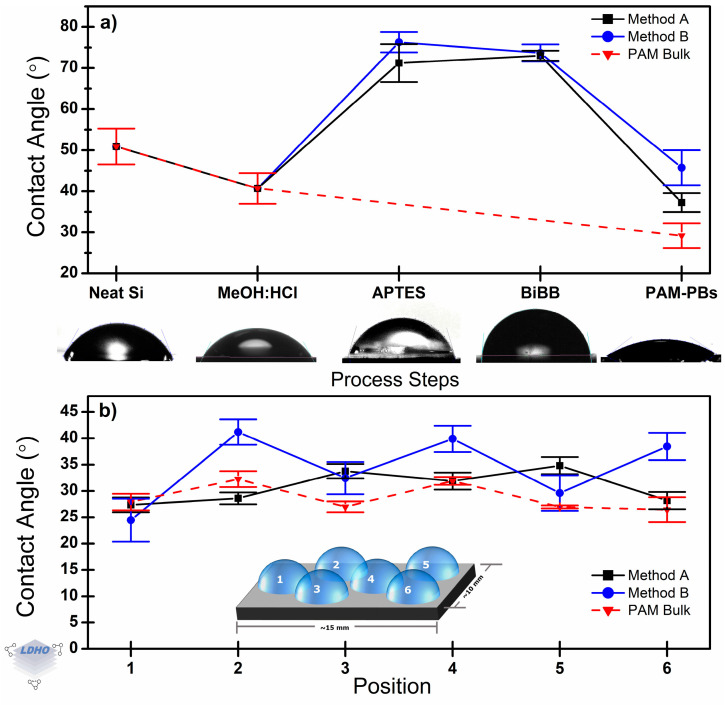
Contact angle measurements for (**a**) Method A, Method B, and commercial bulk PAM film (40k Mn) along the whole synthesis process with representative droplet images and (**b**) CA measurements for each synthesis method considered in this study over 6 regions distributed on the surface, as illustrated.

CA measurements evaluated the evolution of the surface characteristics along the whole synthesis process (Figure 8a), and surface homogeneity for each method was analyzed (Figure 8b). It is well-known that PAM is a hydrophilic material; this was corroborated by the low CA value measured (29.2° ± 3.0°) on a thin film of bulk PAM, which was spin-coated over neat silicon substrates. It will be considered an interesting reference for further discussions. Note that this CA value is ~22° below that corresponding to the neat substrate (50.9°), which confirms that the thin film is effectively covering the substrate. As previously reported [63,68], in bulk PAM material, the surface functional groups are in contact with water during CA measurements to become hydrated, creating hydrogen bonds and polar attraction. However, while the hydrophilic nature was kept, grafting Method A and Method B did not exhibit the same CA value and even had a difference between themselves (by 8.5°, on average). Furthermore, it was observed that Method A presented a different surface coverage as compared to Method B, which could be correlated to potential differences in polymer chain grafting density.

After Cu^0^-SI-ATRP brush polymerization, the CA value for materials synthesized by Method A was 37.3° ± 2.3°, which is lower than that for Method B (45.8° ± 4.3°). These two CA values permit a discernment of how PAM is deposited depending on the synthesis methods. The structural alignment (generally perpendicular to the substrate would limit the number of functional end-groups in the deposited material that interacts with water molecules during the measurement. Depending on the specific PAM (or PAM-PBs)—water interaction, differences in water absorption and further polymer swelling can be observed [69,70]. In this manner, CA values are tied to grafting density. A CA closer to the bulk material may correlate to a higher grafting density by a denser surface coverage over a higher CA value.

To evaluate the surface distribution of the deposition, six equidistant test points for every sample were analyzed by CA measurements, as per the inset in Figure 8b. Through the whole sample, CA values showed a variation of 1.5° ± 0.2° when Method A was used, and for Method B, it was 3.0° ± 0.6°. This supports what was stated above from the FTIR analysis about a more effective surface coverage when Method A is used. CA values follow the order bulk PAM < Method A < Method B. This could suggest that the grafted density and thin film formation by Method A may lead to a densely packed material (high graft density) with surface properties closer to the bulk material. According to the Wenzel-type wetting model, the intermolecular distance between adjacent polymer chains allows water infiltration when the material is synthesized from Method A, but due to the relatively higher brush density, the observed surface response is mainly attributed to PAM-PBs—water interaction. In contrast, if there is a reduced surface density of brushes, as is proposed in the case for Method B, then infiltration may be a dominant behavior, but as brushes are of a nanoscale (~150 nm) dimension, then a droplet of 5 μL would have sufficient volume to saturate the brush film and contact the substrate, allowing for the substrate properties to come into effect. This is observed in Figure 8b, where CA measurements showed variation in their values of up to 7.8°, 16.8°, and 4.9° when Method A, Method B, and bulk PAM deposition were considered, respectively. This could be a consequence of the surface preparation parameters, improving effective area coverage, structure, and orientation of brush formation.

CA proves to be sufficient for a quick and efficient characterization tool both to confirm surface modification and evolution across the PBs synthesis steps. Additionally, it was observed that CA measurements could highlight differences in surface coverage or graft density as Method A and Method B had different CA values from each other in spite of both being Cu^0^-SI-ATRP methods. This is further emphasized by the difference in general CA values between a bulk material thin film and Cu^0^-SI-ATRP methods, marking an intrinsic difference. Therefore, despite bulk PAM and PAM-PBs having the same functional groups (as discussed in FTIR analysis), their structure presents unique and different properties depending on the synthesis route, as observed through CA measurements.

## 4. Conclusions

PAM-PBs were synthesized through the Cu^0^-SI-ATRP method. Through the step-by-step analysis across the different stages of the synthesis route, the impact, relevance, and dependence (or independence) of some parameters were identified. The analyzed parameters allowed a full understanding, as much as possible, of the Cu^0^-SI-ATRP synthesis method, starting from surface preparation.

Surface activation plays an important role in ensuring surface distribution and successful film formation, as it enables favorable molecular groups on the substrate surface that will be used for the subsequent layer-by-layer deposition. As evidenced by FTIR measurements, the use of an ultrasonic bath favored the desired functional group availability on a silicon surface, as further corroborated after APTES deposition. Bands associated with siloxane groups at 1139 cm^−1^ and 1026 cm^−1^ shifted to 1115 cm^−1^ and 1031 cm^−1^ and gained sharpness. Amine bands at 1651 cm^−1^ and at 1531 cm^−1^, respectively, had a reduced *primary_A_1651_*/*secondary_A_1531_* amine ratio of 1.2 in comparison to the *primary_A*/*secondary_A* amine ratio (=2.1) obtained when no mixing method was used. This is further contrasted by the dominant amine band –C–N (~1233 cm^−1^), which has a relative intensity difference of 87% (*primary_A_1651_*/*secundary_A_1233_* = 7.7). Furthermore, silanol bands were found to accentuate a hydrolyzation orientation, as asymmetric stretching band ~1115 cm^−1^ could be the indicator for lateral or hydrolyzed APTES orientation, with ~1030 cm^−1^ siloxane band confirming tethering, and the silanol shoulder ~980 cm^−1^ indicating surface silanol availability and presence. This work proposes that a *siloxane*/*silanol* ratio ≥ 1.5 could be an indicator to determine APTES attachment, with a higher ratio indicating hydrolyzation confirmation, as was observed. This band analysis considers APTES hydrolyzation to be favorable as it promotes homogenous film depositions.

APTES layer formation can be promoted even at the lower precursor solution concentration considered in this study (1 Vol%). Additionally, it was confirmed that the use of mixing methods hindered APTES anchorage. Upon APTES deposition, drying can be omitted, as BiBB anchorage was confirmed and manifested beneficial bands from the C–Br group at 1170 cm^−1^, methyl bands from 3000–2800 cm^−1^, and dominant amine bands around 1550 cm^−1^. It was found that BiBB and its aggressive by-products of the bromide-base serve as self-regulating mechanisms that cleave excessive APTES to ensure both BiB– availability and amine groups (activated amines) for anchoring the initiator molecule. This effect was later confirmed when analyzing BiBB anchorage.

BiBB solutions and anchorage did not show sensitivity to the concentration of the precursor solution. Mixing methods offered the most noticeable changes; ultrasonication seemed to benefit the reactions incurred from solution esterification, causing starch formation. Additionally, it is proposed that the C–Br band correlates to the BiBB molecule and is tied to BiBB anchorage to confirm end-group fidelity. This band was found to not be discernible when no mixing was used, while the US method manifested a shoulder at 1177 cm^−1^. This is further enhanced by premixing the mixture for 5 min prior to the incorporation of substrates instead of continuously mixing the solution. Particularly, uncommon tertiary amide bands at 1558 cm^−1^ called attention to the amine bands centered at 1596 cm^−1^ as it could suggest a higher density of activated sites, as compared to the “continuous mixing” method. As such, IR spectroscopy could identify changes, with BiBB being mostly defined by the amine-amide group changes, an increase in the prevalence of methyl groups, the manifestation of a tertiary amine group, and the manifestation of the C–Br band ~1170 cm^−1^.

PAM-PBs synthesized by Cu^0^-SI-ATRP were found to be sensible to monomer concentration and reaction time. Reducing AM concentration to half of the original value (0.17 M) preserves brush formation, as evidenced by consistent FTIR bands for amide (1645–1603 cm^−1^) and methyl (2923–2856 cm^−1^) groups, mirroring commercial PAM. However, further reduction to 0.09 M disrupts polymerization uniformity, causing shifts in primary amide toward the amine region at 1579 cm^−1^ and tertiary amine that manifested at 1530 cm^−1^, indicative of possible branching or crosslinking. Similarly, synthesis time reveals a narrow operational window from 15–30 min that yields well-defined PAM brushes, while exceeding 60 min degrades structural integrity, marked by altered *carbonyl_Am*/*primary_Am* amide band ratios (1.7 vs. 1.2 at 30 min) and could serve as evidence for conformation changes. Balancing Cu^0^-SI-ATRP parameters and conditions may lead to further advances in polymerization efficiency and brush fidelity, ensuring reproducible, high-quality PAM brush synthesis for advanced surface applications.

Method A, the Cu^0^-SI-ATRP method for PAM-PBs developed here, showed similar spectra to Method B (which was based on the literature). Both methods shared spectra bands that were distinctive for PAM when compared to a commercial-bought bulk PAM of 40k Mn. Moreover, statistical analysis magnified the differences between Method A and Method B in their molecular group contributions. It would seem Method A presents greater reproducibility, whereas in Method B, when peak positions are determined, the higher standard deviations suggest structural irregularities, such as branching or crosslinking. This could explain the differences observed between methods in CA measurements, where surface modification and evolution were successfully observed, as CA also offered a complementary insight.

Contact angle (CA) measurements revealed distinct wettability behaviors between bulk PAM and synthesized PAM-PBs—obtained from Method A and Method B. Bulk PAM exhibited high hydrophilicity (CA: 29.2° ± 3.0°), attributed to its dense hydration via polar amide groups. In contrast, Cu^0^-SI-ATRP-grafted brushes showed intermediate contact angles, with Method A (37.3° ± 2.3°) being closer to the bulk material than Method B (45.8° ± 4.3°) in hydrophilicity and surface homogeneity. The reduced CA variability in Method A (1.5° ± 0.2° vs. 3.0° ± 0.6° for Method B) suggests higher grafting density and uniform surface coverage, mimicking bulk-like behavior. This work proposes that structural differences are responsible for these differences, with Method A having densely packed, perpendicularly aligned brushes that limit substrate interaction. We propose water infiltration through the intermolecular spaces of brushes causes a wetting state by which surface tension creates a contact boundary at the brushes, while Method B’s irregular grafting permits substrate influence by having larger intermolecular spaces, elevating CA. This difference from bulk PAM calls attention to structure and possible conformation, as PAM-PBs seem to balance hydration and grafting density, whereas bulk PAM exhibits its volumetric hydrophilicity. Additionally, Method A had a hydrophilic behavior closer to the bulk material while presenting a more effective surface coverage, highlighting not only an improvement over Method B but also that CA is a property that can be influenced by synthesis parameters. This can be further explored by future works, as graft density differences can be attributed to the differences between the CA measurement of Method A and Method B. These findings highlight CA as a sensitive indicator of surface quality and synthesis efficacy and prove to be sufficient in differentiating brush-based thin films from bulk thin films.

Collectively, these findings place CA and FTIR as quick and efficient methods by which PAM-PBs can be characterized, identified, and confirmed. Additionally, because Method B, a literature-based method, is flexible and capable of allowing brush growth with other monomers, and due to its similarity with Method A, other monomers can be used to benefit from the increased reproducibility and surface uniformity reported in our work.

## Data Availability

The datasets presented in this article are available from the first author (or corresponding author) under request.
